# Shifts in retinal vessel diameter and oxygen saturation in Chinese type 2 diabetes mellitus patients

**DOI:** 10.1186/s12886-016-0217-1

**Published:** 2016-04-19

**Authors:** Yanmin Dong, Leilei Lin, Hong Yan, Yue Fu, Yao Zong, Yongguang Yuan, Xia Huang, Yujie Li, Huining He, Qianying Gao

**Affiliations:** State Key Laboratory of Ophthalmology, Zhongshan Ophthalmic Center, Sun Yat-sen University, 54S Xianlie Road, Guangzhou, 510060 China

**Keywords:** Vessel diameter, Oxygen saturation, Retinal oximeter, Diabetes mellitus, Diabetic retinopathy

## Abstract

**Background:**

The aim of this study was to analyze the shifts in retinal vessel diameter and oxygen saturation in diabetic patients with and without diabetic retinopathy (DR), as well as to assess the association between diabetes duration and either vessel diameter or oxygen saturation.

**Methods:**

In total, 99 Type 2 DM patients were recruited for the study and were divided into three groups: DM with non-obvious retinopathy (DM, *n* = 29), non-proliferative diabetic retinopathy (NPDR, *n* = 40), and proliferative diabetic retinopathy (PDR, *n* = 30). In addition, 78 age-matched healthy individuals were chosen as the control. The diameter and oxygen saturation of the retinal vessels were analyzed using a noninvasive retinal oximeter, and then compared between the three groups and the normal control. Association analysis was applied to analyze the possible influencing factors, including the diameter and oxygen saturation of retinal vessels, on best corrected visual acuity BCVA, as well as the relationship between diabetes duration and the oximetry values.

**Results:**

All of the diabetic patients showed thinner arterioles, wider venules, and a smaller arteriolar-to-venular ratio (AVR) than the healthy individuals. The AVR results from the controls through to the PDR group were 0.81 ± 0.07, 0.78 ± 0.07, 0.76 ± 0.07 and 0.67 ± 0.07, respectively. Both the NPDR and PDR groups showed significantly smaller AVR than the control. All of the diabetic patients exhibited higher retinal vessel oxygen saturation than the healthy individuals. Among all of the oximetry values, AVR exhibited the most significant correlation with best corrected visual acuity (BCVA) (β = 1.533, *P* < 0.0001). An increased diabetes duration was associated with decreased arteriolar diameter (slope = −0.082 pixels/year, *r*^2^ = 0.085, *P* = 0.004) and AVR (slope = −0.009/year, *r*^2^ = 0.349, *P* < 0.001), and with increased venular diameter (slope = 0.104 pixels/year, *r*^2^ = −0.109, *P* = 0.001).

**Conclusions:**

In this Chinese population with type 2 DM, the thinner arterioles and wider venules point to microvascular dysfunction in DR. The increased oxygen saturation of the retinal vessels suggests that retinal oxygen metabolism is affected in diabetic retinopathy.

## Background

Diabetes mellitus (DM) is a global disease that does not only concern aged persons. According to estimates by the World Health Organization, the number of people worldwide with DM is expected to rise to approximately 360 million by 2030 [1]. Further, Type 2 diabetes (T2 DM) has now spread to almost every country and region in the world. China has enjoyed rapid economic development over recent decades. However, this development has also resulted in the increasing prevalence of overweight and obesity, which inevitably drives the diabetes epidemic [2–5]. It has been estimated that 9.6 per 1000 person-years in men and 9.2 in women are subject to T2 DM in China [6].

Diabetic retinopathy (DR) is one of the most common and indeed most severe microvascular complications of DM. It has been shown that the disease is associated with early retinal vascular dysregulation. Also, in the latter stages of the disease, retinal tissue hypoxia is a major trigger of sight-threatening neovascularization. It is therefore important to assess the retinal vascular diameter and retinal oxygenation status of DM patients in order to gain insight into the progression of DR.

Previous studies have primarily focused on the association between retinal vascular calibers and the risk of diabetes or DR. Multiple studies showed that the incidence of both diabetes and DR were associated with narrowing arteriolar [7, 8], wider venular [9, 10], and a smaller arteriolar-to-venular ratio (AVR) [7, 11]. Additionally, Kifley et al. found the increasing severity of DR in persons with diabetes to be associated with a widening of the retinal venular caliber [10]. However, data from the Wisconsin Epidemiologic Study of Diabetic Retinopathy (WESDR) showed that neither retinal arteriolar nor venular calibers as measured at baseline were associated with the incidence or progression of DR [12].

It is certain that hypoxia plays an important role in the pathophysiology of diabetes. Previous studies utilizing oxygen-sensitive microelectrodes have demonstrated that retinal hypoxia exists in the process of diabetes [13, 14]. Additionally, several studies using a noninvasive retinal oximeter found increasing oxygen saturation of the retinal vessels in diabetes [15–18], which indirectly proved retinal hypoxia. Also, Khoobehi and colleagues identified a trend of increasing retinal oxygen saturation from the controls to the NDR group, pointing to increasing levels of DR [17].

Most previous studies have focused on retinal vascular parameter or retinal vessel oxygen saturation separately, and the research subjects were mostly Caucasians. In our study, we analyzed the retinal vessel diameter and vessel oxygen saturation of T2 DM patients with and without retinopathy in China in order to detect shifts associated with the severity level of diabetes compared with healthy individuals. This study was also performed to detect the impacts of vessel diameter and oxygen saturation on visual acuity, as well as to assess the relationship between diabetes duration and either vessel diameter or oxygen saturation. The aim was to identify a more sensitive and noninvasive method for evaluating the severity and prognosis of diabetes.

## Methods

The study protocol was reviewed and approved by the Medical Ethics Committee of the Zhongshan Ophthalmic Center, Sun Yat-sen University (No.2013MEKY028). It also strictly adhered to the principles of the World Medical Association Declaration of Helsinki. All subjects signed informed consent forms prior to participation.

### Subjects

A total of 99 Type 2 DM patients were recruited from the outpatient clinic at Zhongshan Ophthalmic Center. Patients were excluded if they had obvious cataract or other media opacities, optic nerve disease, other retinal diseases except DR, intraocular pressure (IOP) >21 mmHg, or had previously undergone laser photocoagulation. In addition, 78 age-matched healthy persons were recruited as the control group. The exclusion criteria for the control group were any kind of systemic disease, any history of ocular disease, trauma, or eye surgery, current pregnancy, and breast-feeding. Only the right eye of each subject was used for the analysis. The 99 right eyes of the diabetes patients were divided into three groups: Group 1: DM with non-obvious retinopathy (DM, *n* = 29), Group 2: non-proliferative diabetic retinopathy (NPDR, *n* = 40), and Group 3: proliferative diabetic retinopathy (PDR, *n* = 30). The three groups were constituted according to the international criteria formulated in 2002 [19].

All subjects answered a standardized questionnaire about their duration of diabetes, history of ocular and systemic conditions, and medication use. The basic examinations involved: best corrected visual acuity (BCVA, logMAR visual acuity chart), intraocular pressure (Canon TX-20, Canon Corporation, Tokyo, Japan), slit-lamp examination (Suzhou YZ5S, Suzhou Liuliu, China), systolic blood pressure (BPsyst) and diastolic blood pressure (BPdiast), heart rate (BangPu, BF-1100, Shenzhen BangPu Corporation, Shenzhen, China), and finger pulse oximetry (Biolight M70, Biolight Corporation, Zhuhai, China). Further, the mean ocular perfusion pressure (OPPm) driving blood through the retina is calculated using the following equation [20]:

$$ OPPm=\frac{2}{3}\left[ BPdiast+\frac{1}{3}\left( BPsyst- BPdiast\right)\right]-IOP. $$

The basic information of subjects studied is detailed in Table 1.

**Table 1:** Clinical data and retinal oximetry values of the groups studied

Group	Normal (*n* = 78)	DM, no DR (*n* = 29)	NPDR (*n* = 40)	PDR (*n* = 30)
Age	55.59 ± 7.88	58.79 ± 10.08	60.53 ± 6.76	55.83 ± 6.66
Sex (M/F)	28/50	14/15	15/25	16/14
Finger_satO_2_	97.30 ± 1.27	97.35 ± 1.08	96.98 ± 4.95	97.50 ± 0.98
OPPm	49.42 ± 7.71	51.06 ± 6.59	52.75 ± 9.47	54.57 ± 8.60
BCVA	0.78 ± 0.28	0.64 ± 0.27	0.45 ± 0.30	0.28 ± 0.21
Diabetes-duration (yrs)	-	6.59 ± 4.12	8.9 ± 5.49	11.93 ± 4.65
A_diameter (pixels)	13.47 ± 1.19	13.2 ± 1.12	13.03 ± 1.46	11.82 ± 1.47**
V_diameter (pixels)	16.80 ± 1.68	17.0 ± 1.62	17.28 ± 1.84	17.66 ± 1.44*
AVR	0.81 ± 0.07	0.78 ± 0.07	0.76 ± 0.07**	0.67 ± 0.07**
A_SatO_2_(%)	95.00 ± 4.78	97.45 ± 5.76	100.02 ± 7.48**	112.56 ± 10.41**
V_SatO_2_(%)	58.50 ± 3.76	61.48 ± 7.52	65.98 ± 6.23**	68.06 ± 6.00**
AV_difference (%)	36.49 ± 4.35	35.97 ± 6.41	34.03 ± 6.26	44.5 ± 10.07**

BCVA was recorded by equivalent decimal values. Diameter conversion factor: 1 pixel ≈ 9.3 μm

*p < 0.05, **p < 0.01

### Retinal oximetry

The noninvasive retinal oximeter Oxymap T1 (Oxymap, Reykjavik, Iceland) has been described previously [21, 22]. Briefly, it is an add-on to the fundus camera (Topcon TRC-50DX; Topcon Corporation, Tokyo, Japan), which combines spectroscopy and multispectral imaging techniques. The Oxymap Analyzer software analyzes the images from the oximeter and automatically returns the relative oxygen saturation and vessel diameter. The method used for the vessel diameter measurements and oxygen saturation calculation has been described previously, and the results proved reliable and reproducible [21, 23].

### Imaging and analysis

**Fig. 1:**
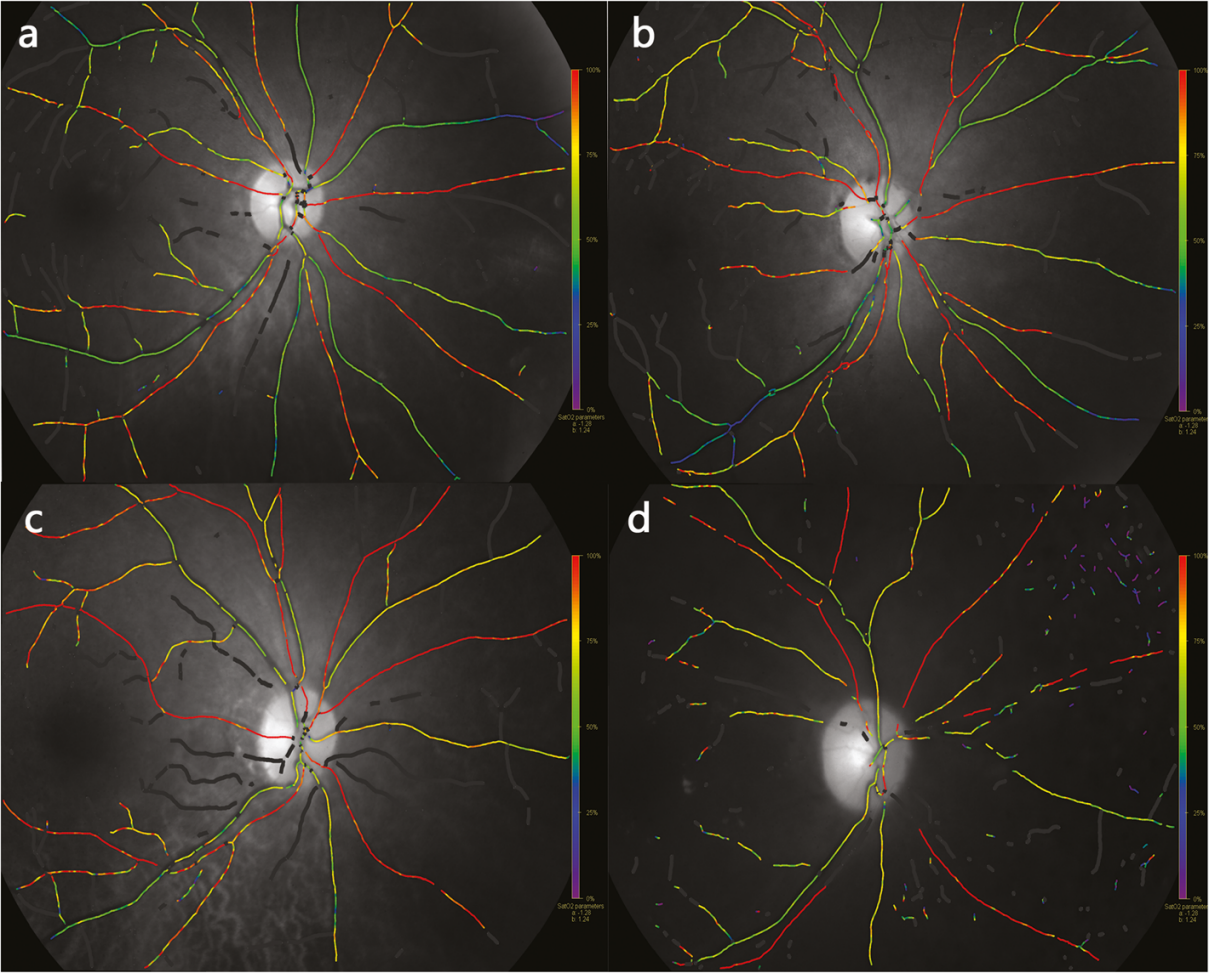
Pseudocolor fundus maps of healthy individual and diabetic patients with various severity: DM with no obvious DR, NPDR and PDR. **a** A healthy 58 yrs female: A_SatO_2_ = 95.26 %, V_SatO_2_ = 57.54 %, A_diameter = 13.14 pixels, V_diameter =15.24 pixels. **b** A 60 yrs femal, DM patient with no obvious DR: A_SatO_2_ = 97.47 %, V_SatO_2_ = 63.17 %, A_diameter = 12.34 pixels, V_diameter = 15.49 pixels. **c** A 56 yrs femal, NPDR patient: A_SatO_2_ = 99.0 %, V_SatO_2_ = 69.61 %, A_diameter = 13.56 pixels, V_diameter = 17.04 pixels. **d** A 52 yrs male PDR patient: A_SatO_2_ = 115.73 %, V_SatO_2_ = 69.67 %, A_diameter = 11.56 pixels, V_diameter = 18.51 pixels

The pupils were dilated with 0.5 % tropicamide (Shenyang Xingqi Corporation, Shenyang, China). All of the fundus images were taken in a dark room and were performed by the same skilled photographers according to consistent parameters. All of the subjects were examined twice, and all of the images were centered on the optic disc, with about one minute’s space between images. Further, the best quality image was selected for analysis (Fig. 1).

The pseudocolor fundus images were analyzed according to a standard protocol. The optic disc was first excluded by a circle to avoid highly reflective background. Then, a second circle of three times the optic disc radius was created, and the vessel segments between the two circles were selected for analysis. The mean oxygen saturation and mean width of the selected retinal arteries and veins were automatically analyzed by Oxymap Analyzer version 2.4, a specialized software.

### Statistical analysis

The statistical analysis was performed using the R software package, version 3.1.3 (The R Foundation for Statistical Computing, available at http://www.R-project.org/). The Kruskal-Wallis test was applied to test for differences in the diameter of the retinal arterioles (A_diameter) and venules (V_diameter), and the AVR, as well as the oxygen saturation of the retinal arterioles (A_satO_2_) and venules (V_satO_2_), and the arteriole-venule difference (AV_difference) between the groups. All data was expressed as mean ± standard deviation (SD). For all of the analyses, *P* < 0.05 was considered statistically significant.

Meanwhile, a univariate analysis was applied to analyze the possible influencing factors, including the diameter and oxygen saturation of the retinal vessels, on BCVA. The multivariate analysis of the oximetry values included: diabetes duration, age, sex, finger pulse SatO_2_ and OPP. The ocular perfusion pressure was derived from the BPsyst, BPdiast, and IOP, so we chose OPP instead of utilizing all three.

## Results

### Vessel diameter

**Fig. 2:**
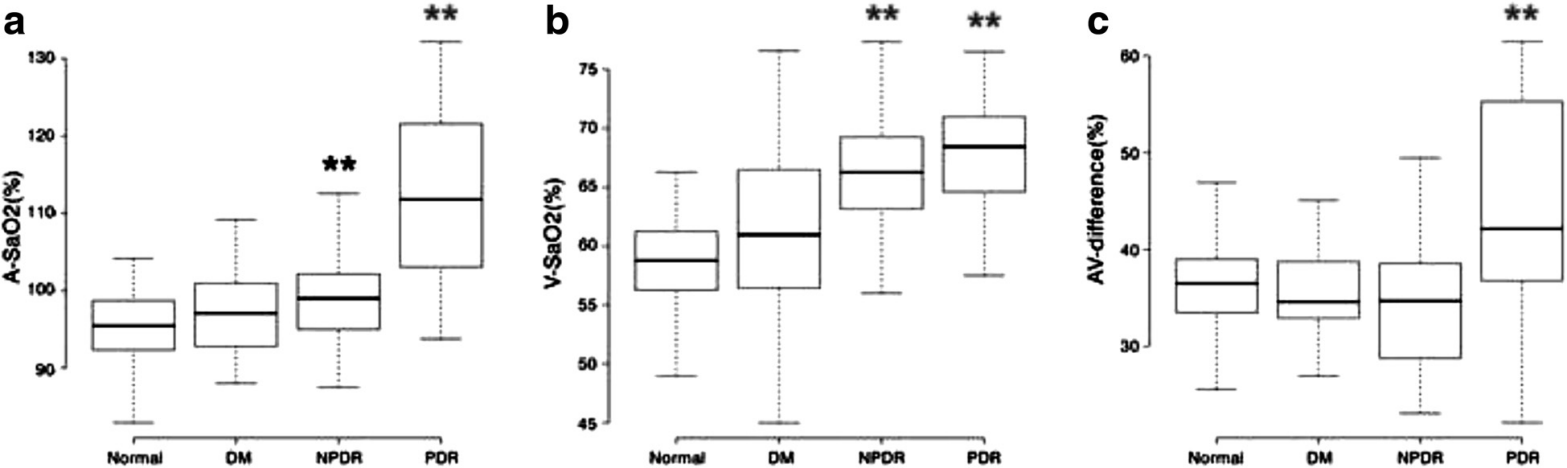
The diameter of retinal arterioles and venules, AVR in the four groups studied. All diabetic patients showed thinner arterioles, wider venules, and smaller AVR compared with normal control, which varied with severity from DM with no DR to PDR. (**p < 0.01, *p < 0.05)

In healthy individuals, the diameters of the arterioles and venules were13.47 ± 1.19 pixels, and 16.80 ± 1.68 pixels, respectively, and the AVR was 0.81 ± 0.07. All diabetic patients showed thinner arterioles and wider venules and, therefore, smaller AVR than the healthy individuals, which changed according to the severity of the disease. Only the PDR group exhibited statistically significant thinner arterioles and wider venules when compared with the normal group. However, both the NPDR and PDR patients had significantly smaller AVR than the controls (*p* < 0.01; Fig. 2).

The vessel diameter values are listed in Table 1.

### Oxygen saturation

**Fig. 3:**
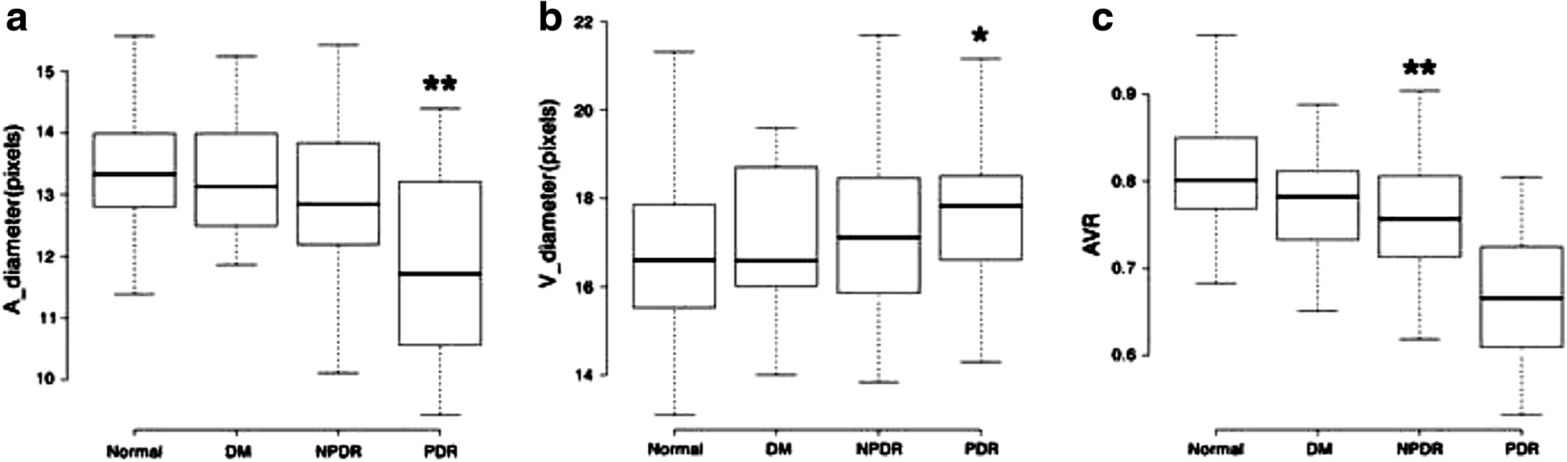
The oxygen saturation of arterioles, venules and AV-difference in the four groups studied. Both arteriolar and venular oxygen saturation showed increasing trend with increasing severity of disease (**p < 0.01)

The retinal oxygen saturation in healthy individuals was 95.0 ± 4.78 % in the arterioles and 58.50 ± 3.76 % in the venules. Compared with the normal control group, all of the diabetes patients showed higher oxygen saturation in both the arterioles and venules. Further, the differences between the NPDR or PDR patients and the normal controls were both statistically significant (*p* < 0.01; Fig. 3). Additionally, there was an obvious increasing trend in either arteriolar or venular oxygen saturation with the increasing severity of disease, although significance was only reached for the comparison of controls to the NPDR and PDR groups.

The AV difference was 36.49 ± 4.35 % in healthy individuals, and the corresponding values for groups 1 to 3 were 35.97 ± 6.41 %, 34.03 ± 6.26 %, 44.5 ± 10.07 %, respectively. There was no significant difference between the diabetes patients, except for the PDR group and normal controls.

The oxygen saturation values are listed in Table 1.

### Associations analysis

#### Associations between oximetry values and BCVA

**Fig. 4:**
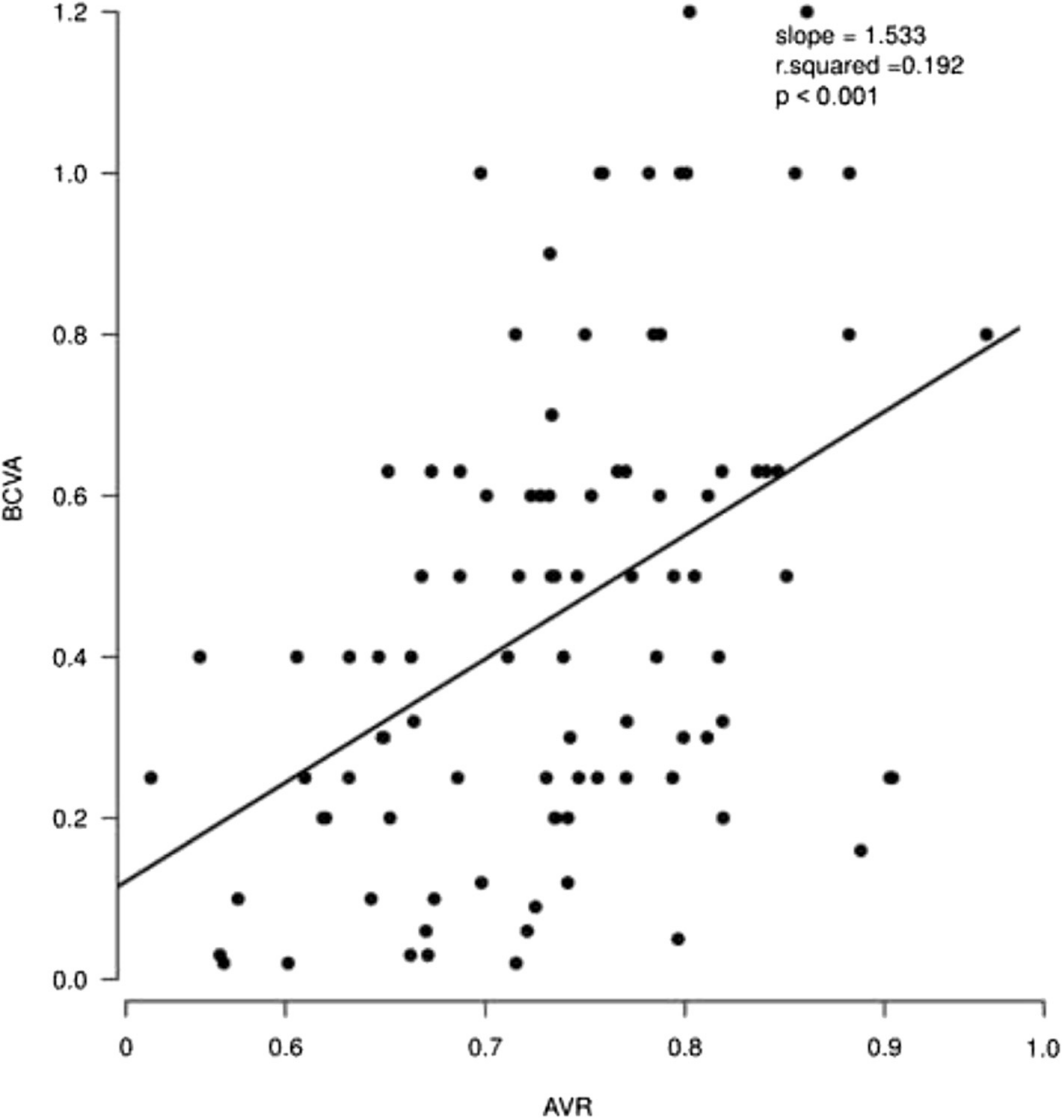
Associations between AVR and BCVA. Larger AVR was correlated with better BCVA. (BCVA: decimal values)

Besides the V_diameter, all of the oximetry values were found to be associated with BCVA (Table 2). Higher A_SatO_2_, V_SatO_2_, and AV_difference were found to be correlated with lower BCVA. Conversely, both wider arterioles and larger AVR were correlated with better BCVA. AVR exhibited the most significant correlation with BCVA (*p* < 0.001; Fig. 4).

**Table 2:** Associations between oximetry values and BCVA

Parameters	β (95 % CI)	t value	*P* value
(Intercept)	2.08 (1.541 to 2.619)	7.664	<0.001
A_SatO_2_	−0.016 (−0.021 to −0.011)	−6.023	<0.001
(Intercept)	1.205 (0.648 to 1.762)	4.293	<0.001
V_SatO_2_	−0.012 (−0.02 to −0.003)	−2.694	0.008
(Intercept)	0.951 (0.702 to 1.2)	7.596	<0.001
AV_difference	−0.013 (−0.02 to −0.007)	−4.082	<0.001
(Intercept)	−0.236 (−0.743 to 0.271)	−0.925	0.357
A_diameter	0.054 (0.015 to 0.094)	2.719	0.008
(Intercept)	1.016 (0.389 to 1.643)	3.217	0.002
V_diameter	−0.032 (−0.068 to 0.004)	−1.791	0.076
(Intercept)	−0.676 (−1.153 to −0.198)	−2.807	0.006
AVR	1.533 (0.888 to 2.178)	4.72	<0.001

#### Associations between retinal vessel diameter and candidate variables (diabetes duration, age, sex, finger SatO_2_ and OPP)

**Fig. 5:**
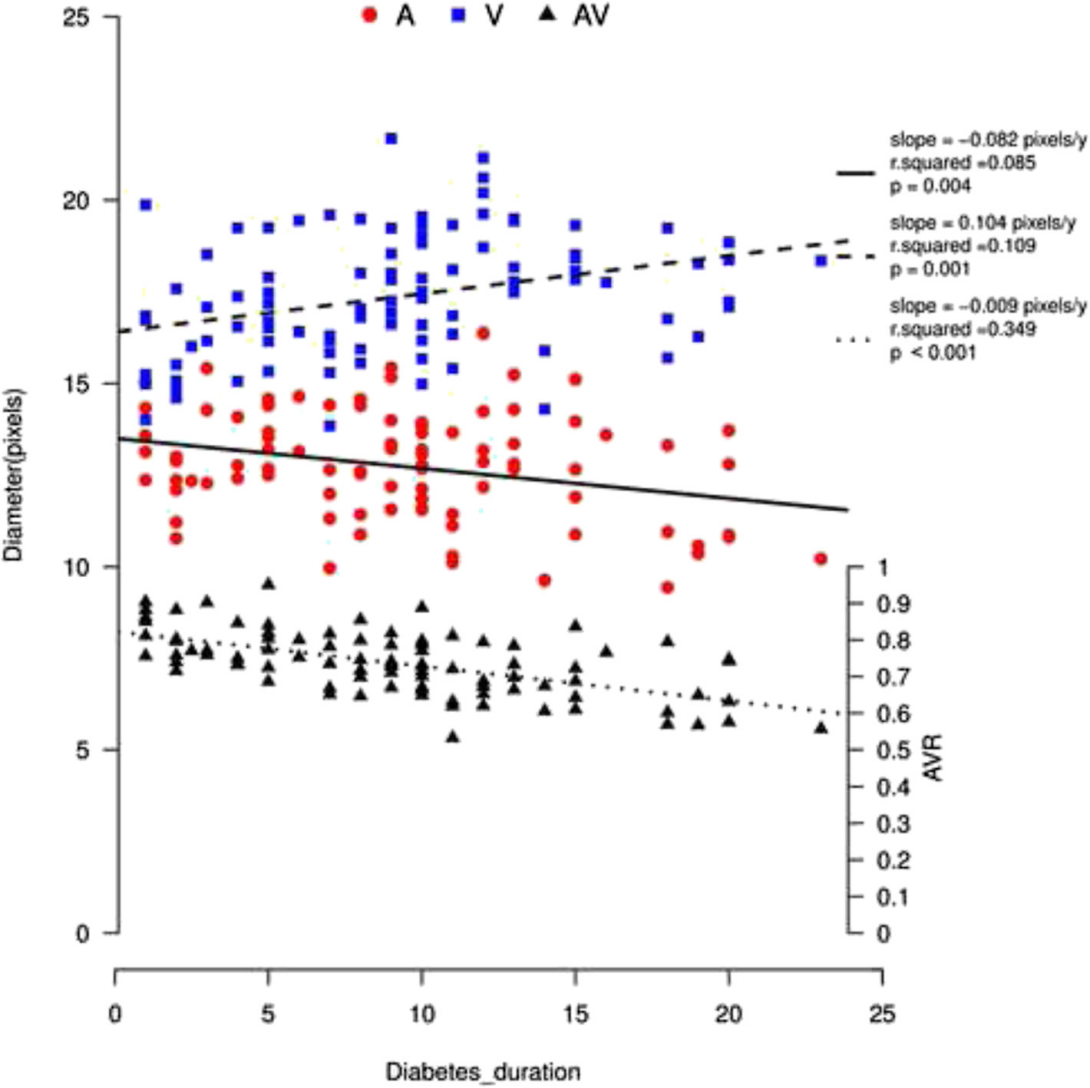
Retinal vessel diameter (pixels; left y-axis) of arterioles (*red dots*) and venules (*blue squares*), and AVR (right y-axis) with increasing diabetes duration (*dark triangles*). There was a significant decrease in A_diameter, an increase in V_diameter, and smaller AVR with increasing diabetes duration

In a separate simple linear regression with retinal vessel diameter in relation to diabetes duration, there was a significant decrease in A_diameter, an increase in V_diameter, and smaller AVR with increasing diabetes duration (*p* < 0.01; Fig. 5). A multivariate analysis using multiple linear regression was also performed with the following variables included: age, sex, finger SatO_2_ and OPP. The shift trends in A_diameter, V_diameter and AVR with diabetes duration were still significant (Table 3).

**Table 3:** Adjusted associations between retinal vessel diameter and candidate variables

Parameters	β (95 % CI)	t value	*P* value
A-diameter			
(Intercept)	10.041 (1.09 to 18.991)	2.228	0.028
Diabetes_duration	−0.095 (−0.15 to −0.04)	−3.418	0.001
Sex	−0.425 (−0.999 to 0.148)	−1.473	0.144
Age	0.026 (−0.01 to 0.061)	1.451	0.15
Finger_SatO_2_	0.04 (−0.048 to 0.128)	0.898	0.372
OPPm	−0.032 (−0.065 to 0.002)	−1.886	0.062
V-diameter			
(Intercept)	14.093 (3.918 to 24.268)	2.751	0.007
Diabetes_duration	0.101 (0.038 to 0.163)	3.188	0.002
Sex	−0.465 (−1.117 to 0.186)	−1.418	0.16
Age	−0.016 (−0.057 to 0.024)	−0.81	0.42
Finger_SatO_2_	0.031 (−0.07 to 0.131)	0.606	0.546
OPPm	0.009 (−0.028 to 0.047)	0.496	0.621
AVR			
(Intercept)	0.687 (0.271 to 1.104)	3.281	0.001
Diabetes_duration	−0.01 (−0.013 to −0.008)	−7.87	<0.001
Sex	−0.006 (−0.032 to 0.021)	−0.423	0.673
Age	0.002 (0.001 to 0.004)	2.769	0.007
Finger_SatO_2_	0.001 (−0.003 to 0.005)	0.655	0.514
OPPm	−0.002 (−0.004 to −0.001)	−2.982	0.004

#### Associations between retinal vessel SatO_2_ and candidate variables (diabetes duration, age, sex, finger SatO_2_ and OPP)

**Fig. 6:**
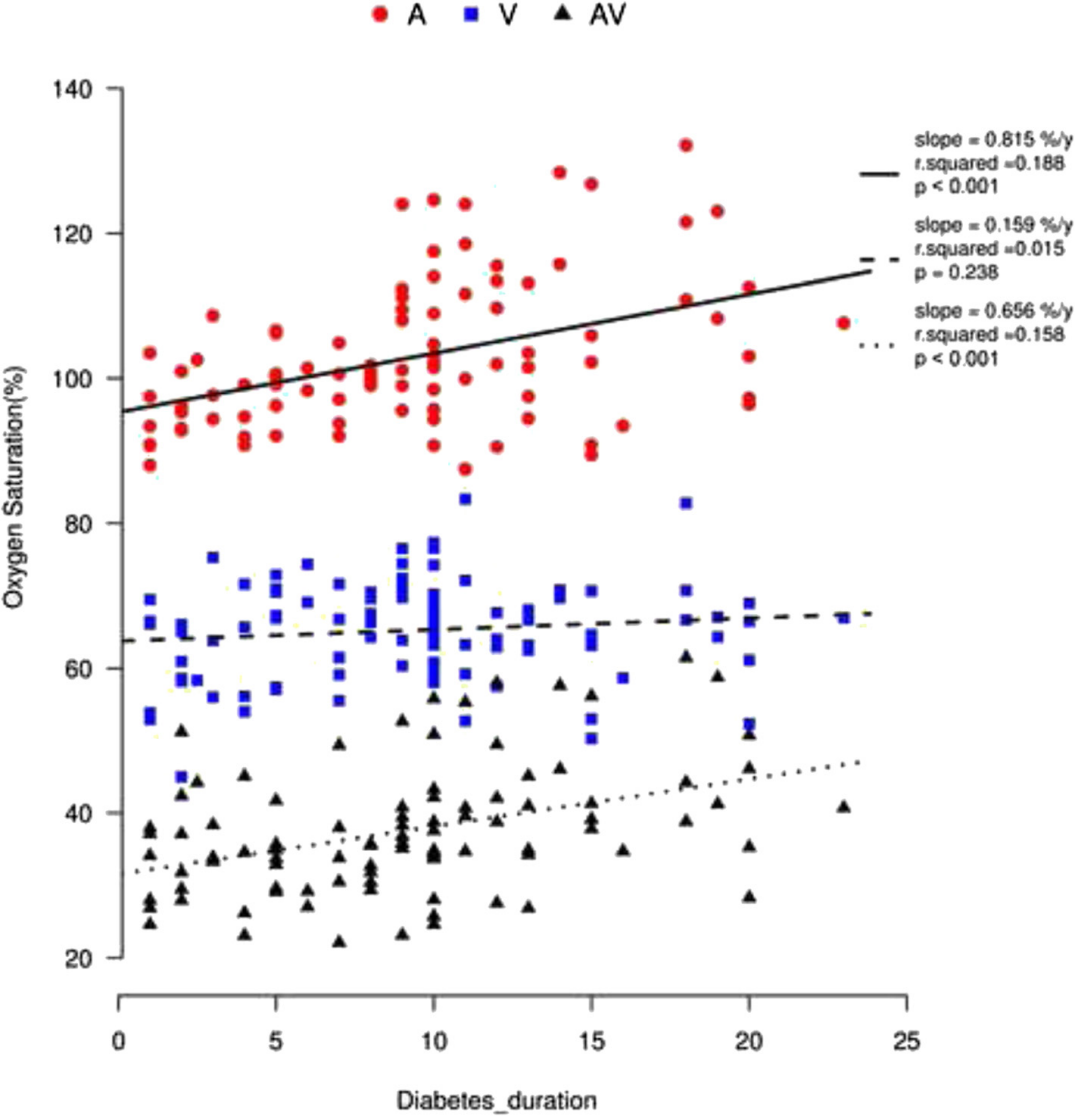
Oxygen saturation (%) with diabetes duration in retinal vessels: arterioles (*red dots*), venules (*blue squares*), and AV difference (*dark triangles*). There was a significant increase in A_SatO_2_ and AV_difference with increasing diabetes duration

In a separate simple linear regression with retinal oxygen saturation in relation to diabetes duration, there was a significant increase in arteriolar oxygen saturation with increasing diabetes duration and a similar trend in AV_difference (*p* < 0.01; Fig. 6). A multivariate analysis using multiple linear regression was performed with the following variables included: age, sex, finger SatO_2_ and OPP. The increases in A_SatO_2_ and AV_difference with diabetes duration were still significant (Table 4).

**Table 4:** Adjusted associations between retinal vessel SatO_2_ and candidate variables

Parameters	β (95 % CI)	t value	*P* value
A-SatO_2_			
(Intercept)	85.937 (25.436 to 146.439)	2.821	0.006
Diabetes_duration	0.818 (0.445 to 1.19)	4.359	<0.001
Sex	−0.608 (−4.484 to 3.267)	−0.312	0.756
Age	−0.018 (−0.257 to 0.222)	−0.146	0.885
Finger_SatO_2_	0.031 (−0.566 to 0.629)	0.104	0.917
OPPm	0.149 (−0.077 to 0.374)	1.31	0.193
V-SatO_2_			
(Intercept)	41.775 (−0.62 to 84.17)	1.957	0.053
Diabetes_duration	0.1 (−0.162 to 0.361)	0.757	0.451
Sex	−4.41 (−7.126 to −1.695)	−3.226	0.002
Age	−0.062 (−0.23 to 0.106)	−0.73	0.467
Finger_SatO_2_	0.232 (−0.187 to 0.65)	1.099	0.275
OPPm	0.106 (−0.052 to 0.263)	1.328	0.187
AV-difference			
(Intercept)	44.162 (−7.697 to 96.021)	1.691	0.094
Diabetes_duration	0.718 (0.399 to 1.038)	4.466	0
Sex	3.802 (0.48 to 7.124)	2.273	0.025
Age	0.044 (−0.161 to 0.25)	0.427	0.67
Finger_SatO_2_	−0.2 (−0.712 to 0.312)	−0.778	0.439
OPPm	0.043 (−0.15 to 0.236)	0.443	0.659

## Discussion

Our study showed that the increasing severity from DM with no DR to PDR was accompanied by thinner arterioles and wider venules, although significance was only reached for the comparison of the normal controls to the PDR group. However, both the NPDR and PDR groups showed significantly smaller AVR than the controls. All of the diabetic patients exhibited higher retinal vessel oxygen saturation than healthy individuals。Also, there was an obvious increasing trend in either arteriolar or venular oxygen saturation with an increasing severity of disease. In addition, we found that AVR exhibited a significant correlation with BCVA. In the study, we also found that the duration of diabetes was significantly associated with the retinal vessel diameter and oxygen saturation.

The retinal vessels offer a unique and easily accessible window through which to study human microcirculation in health and disease. As an important physiological parameter, the retinal vessel diameter has been found to alter in various diseases [24–27], as well as to change with age and exercise intensities [28, 29]. Researchers have proposed retinal vessel diameter as a biomarker with which to determine the risk and progress of cardiovascular disease [30–33]. Previous studies also proved the association between retinal vessel width and the risk of diabetes or DR, although the results were inconsistent. Wong et al. found that participants with narrower retinal arteriolar diameters had a higher incidence of diabetes [7]. However, Cheung and colleagues found that persons with diabetes were more likely to have wider arteriolar and venular calibers than those without diabetes, and that subjects with DR had a wider venular caliber than those without retinopathy [34]. In this study, both thinner arterioles and wider venules were observed in all of the diabetic patients, although significance was only reached for the comparison of controls to the PDR group. Also, there was a significant increase in venular diameter, decrease in arteriolar diameter, and smaller AVR with increasing diabetes duration. The main causes of abnormalities in vascular caliber may be dysfunction of the endothelium and the abnormal tone of smooth muscle cells and pericytes. Retinal blood flow is autoregulated by the interaction of myogenic and metabolic mechanisms through the release of vasoactive substances by the vascular endothelium and retinal tissue surrounding the arteriolar wall [21]. Both pericytes and smooth muscle cells represent the myogenic mechanism for the vascular autoregulation of blood flow. They are able to regulate the capillary diameter through both contraction and relaxation. Local vasoactive substances therefore play an important role in regulating the functional dynamics of the vascular wall.

Endothelin-1 (ET-1) is the most potent endogenous vasoconstrictor known. Multiple studies have shown that the endogenous expression of ET-1 is increased in experimental diabetes [35, 36]. Moreover, the ET-1 mRNA level in patients with DR was found to be significantly higher than in those without DR. It was therefore suggested that the ET-1 level is associated with the severity of DR in patients with Type 2 DM [37]. It is known that ET-1 tends to decrease retinal arterial diameter, but has no effect on retinal venous diameter [38]. Thus, we speculate that increased ET-1 may be one of the main causes of the decreasing arteriolar diameter in diabetes patients.

Dilated venular, which is another feature of the microvascular dysfunction of diabetes, was found to be associated with both the severity and duration of diabetes. This finding is in line with previous studies. Kifleyalso et al. found increasing severity of DR to be associated with the widening of the retinal venular caliber [10]. Steel et al. found that both increased DR severity and increased diabetes duration were associated with increased vascular width [39]. One possible explanation for this is the impairment of the vasomotor reaction resulting from the dysfunction of pericytes and smooth muscle cells. Researchers have found that T2 DM reduces retinal vascular vasoconstrictor responses to hyperoxia and retinal vascular vasodilation responses to flicker stimuli [40, 41]. All of these findings proved the microvascular dysfunction of diabetes.

AVR combines the width of the arterioles and the venules together, which may be more valuable for assessing microcirculation status. In association analysis between the oximetry values and BCVA, AVR exhibited the most significant correlation with BCVA. Larger AVR was correlated with better BCVA. However, larger prospective studies to investigate the relevant causal relationships are warranted.

Previous Caucasian studies also found increased retinal arteriole oxygen saturation in DR patients, which is again consistent with our results [17–19]. There are several possible explanations for the elevated retinal arteriolar oxygen saturation in diabetic retinopathy. First, there is a thickening of the vascular basement membrane in diabetes, which directly increases the oxygen transport distance and inevitably hinders oxygen diffusion [42]. Second, there is decreasing blood flow since, as our results, all DR members exhibited decreased arteriole diameters (Table 1). The decreased arteriole diameter will consequentially slow down the blood flow, resulting in the accumulation of metabolites. Third, there is a greater affinity of hemoglobin for oxygen in diabetic patients [43, 44]. This may also help to explain the increased saturation in the retinal venules.

Regarding venular oxygen saturation, all previous studies have found higher values in the DR group than in the normal control group [15–18]. Khoobehi also showed a trend of increasing retinal oxygen saturation from the controls to the DR group, although significance was only reached for the comparison of the controls to the severe NPDR and PDR groups, and to all DR groups [17]. We propose that there are several reasons for this result. First, due to tissue degeneration and cell death, the demand for and consumption of oxygen in the retina is reduced, so the oxygen extraction from the arterioles is decreased, which consequently results in an increasing venule oxygen saturation. Second, the formation of an AV shunt enables the blood to travel directly into the venules without oxygen extraction by the retina tissues. Therefore, the retinal tissues are relatively hypoxic in DR. Previous studies have verified the hypoxic status of the DR retina [13, 45]. It is suggested that the elevation of the venous oxygen saturation is more closely related to the retinal metabolism. Furthermore, it refers to the oxygen supply and consumption of the retina, which might be more indicative of diabetic changes to the capillary system than the blood flow velocity [15].

At present, the results of AV differences in DR are still controversial. Hammer [15], Jørgensen [18], and Man [46] all found a decreased AV difference. Additionally, they all considered the decreased oxygen consumption and neuronal metabolism, the occlusions and obliterations in the capillary bead, and the formation of AV shunt vessels to be the main causes. Hardarson [16] and Khoobehi [17] found a similar result between DR and healthy persons. Our study showed a decreasing trend of AV difference from the normal controls to DM with no DR group, to the NPDR group. A decreasing AV difference means decreasing oxygen extraction by the retinal tissue, which is the result of tissue degeneration. However, a significant increasing AV difference was observed in the PDR group when compared with the normal controls, and a significant difference only could be found in the PDR group when comparing with the controls. The reason for this is that the PDR patients showed significantly increasing arteriole oxygen saturation, with some exceeding by as much as 130 %. However, the values of venule oxygen saturation did not increase as much as the arteriole oxygen saturation did. Further, during the severe stage of DR, the formation of neovascular might promote the leakage of substances into the adjacent tissue from the vessels. All of these factors might distort the results. The change trend of AV difference in pathophysiologic process of DR still need further longitudinal study.

In the diabetes patients with no retinopathy, there were no significant differences in the diameter and oxygen saturation of retinal vessels when compared with the controls. In the earlier stages of diabetes, the microvascular, oxygen delivery, and metabolism were not significantly impaired [47], so the values of oxygen saturation and vessel diameter were nearly normal.

The main limits to the present study were a lack of serum markers, such as blood glucose and glycated hemoglobin (HbA1c), and blood rheology data. At present, the oscillatory potentials of the electroretinogram (ERG) are objective indicators for predicting early slight abnormities and the progression of DR. Future studies should take these into account. Additionally, a longitudinal study of diabetes that focuses on the consecutive shifts would present more valuable information.

## Conclusions

In this Chinese population with type 2 DM, the thinner arterioles and wider venules point to microvascular dysfunction in DR. The increased oxygen saturation of the retinal vessels suggests that retinal oxygen metabolism is affected in diabetic retinopathy. The retinal vessel diameter and oxygen saturation may potentially play a predictive role in determining the risk and progress of DR.

### Availability of data and materials

All the data supporting the findings was contained within the manuscript.

### Ethics and consent to participate

The study protocol was reviewed and approved by the Medical Ethics Committee of the Zhongshan Ophthalmic Center, Sun Yat-sen University (No.2013MEKY028). It also strictly adhered to the principles of the Declaration of Helsinki. All subjects signed informed consent forms prior to participation.

### Consent to publish

Written informed consents for publication of their clinical images were obtained from the individuals. The details were included in the consent form for participation.

## Abbreviations

A_diameter: diameter of retinal arterioles
A_SatO_2_: oxygen saturation of retinal arterioles
AV_difference: arteriole-venule difference
AVR: arteriolar-to-venular ratio
BCVA: best corrected visual acuity
BPdiast: diastolic blood pressure
BPsyst: systolic blood pressure
DM: diabetic mellitus
DR: diabetic retinopathy
IOP: intraocular pressure
NPDR: non-proliferative diabetic retinopathy
OPPm: ocular perfusion pressure
PDR: proliferative diabetic retinopathy
SD: standard deviation
T2 DM: type 2 diabetes mellitus
V_diameter: diameter of retinal venules
V_SatO_2_: oxygen saturation of retinal venules
